# 

**Published:** 1845-05

**Authors:** 


### CONTENTS

OF THE

### MEDICAL EXAMINER.

NEW SERIES—NO. V.—MAY, 1845.

### ORIGINAL COMMUNICATIONS.

Case of communication between the Ventricles of the Heart;—the Aorta originating from both Ventricles. (Communicated by Professor Dunglison,) - - - - - - - - - 261

Autopsy of a boy who was subject to Epileptic Fits, with loss of Speech, arising from mechanical injury of the head. (Communicated by Dr. Elkinton,) 263

Remarkable Case of Spasm of the Glottis, with unusual Symptoms. By Benjamin F. Eppes, of Palestine, Sussex Co., Va. - - - 264

### CLINICAL LECTURES AND REPORTS.

### PHILADELPHIA HOSPITAL,

*Saturday, January* 11, 1845.

Clinic of Professor Dunglison.

Induration of the Cellular Tissue, - - - - - 268

Tremors, - - - - - - - - - - - 270

Pathological Specimens, - - - - - - - - 270

### BIBLIOGRAPHICAL NOTICES.

Lectures on Animal Heat. By Thomas Spencer, M. D., Professor of the Institutes and Practice of Medicine in the Medical Institution of Geneva College, - - - - - - - - 273

Principles of Forensic Medicine. By William A. Guy, M. B. Cantab. Fellow of the Royal College of Physicians; Professor of Forensic Medicine in King's College, London, &c. First American Edition. With Notes and Additions, by Charles A. Lee, M. D. Professor of Materia Medica and General Pathology in Geneva College, &c. &c. - - - - - - - - - - 277

Accidents—Popular directions for their immediate treatment; with Observations on Poisons and their Antidotes. By Henry Wheaton Rivers, M. D., Sugeon to the United States Marine Hospital, Providence, R. I. - - - - - - - - - 280

A Treatise on Poisons, in relation to Medical Jurisprudence, Physiology, and the Practice of Physic. By Robert Christison, M. D., F. R. S. E., Professor of Materia Medica in the University of Edinburgh, Fellow of the Royal College of Physicians, etc. etc. etc. First American, from the fourth Edinburgh Edition. - 281

1. Annual Report of the Managers of the (N. Y.) State Lunatic Asylum, made to the Legislature, January 31st, 1845. - - - - 282

2. Twenty-fourth Annual Report of the Bloomingdale Asylum for the Insane, for the year 1844. - - - - - - - 284

3. The Annual Report of the Eastern Asylum in the city of Williamsburg, Virginia, for 1844. - - - - - - - - 286

4. Annual Report of the Managers of the (Kentucky) Lunatic Asylum, - - - - : - - - - - - - - 287

Twenty-Eighth Annual Report on the state of the Asylum for the Relief of Persons deprived of their Reason. Published by direction of the Contributors. - - - - - - - - 288

The Principles and Practice of Dental Surgery. By Chapin A. Harris, M. D., D. D. S.; Professor of Practical Dentistry and Dental Pathology in the Baltimore College of Dental Surgery; Fellow of the American Society of Dental Surgeons; Member of the Medico-Chirurgical Faculty of Maryland, etc. etc. Second Edition. Revised, modified, and greatly enlarged. Illustrated by sixty-nine wood engravings, - - - - - - - 289

EDITORIAL.

Health of Philadelphia, - - - - - - - - - - 290

Obituary, - - - - - - - - - - - - - - 291

RECORD OF MEDICAL SCIENCE.

Observations on the Nature, Origin, and Treatment of Puerperal Fever, founded on Cases which occurred Epidemically at Plymouth, in the Autumn of 1831; and on a Comparative View of Concurrent Cases of abdominal inflammation in the Puerperal State, the Pregnant State, and after a Miscarriage; and of Cases in Women not Pregnant; of Abdominal Inflammation occurring in various Diseases then prevalent; e. g., Synochus, Cholera, Erysipelas, and a peculiar external Gangrenous Affection; and on a View of Peritoneal Inflammation in the Male Sex. By Edward Blackmore, M. D., Edin, - - - - - - - - 292

Paralysis of the Intestines.—Obstinate constipation of the Bowels, - 301

Caoutchouc as a Remedy for Toothache, - - - - - - - 302

Unusual form of Intus-Susceptio of the Colon, - - - - - - 303

Influenza amongst Horses, - - - - - - - - - - 305

On the Pulsating Tumours of bone; with the account of a case in which a Ligature was placed around the Common Iliac Artery. By Edward Stanley, F. R. S., Surgeon to St. Bartholomew's Hospital, - - - - - - . - - - - - - 306

Surgical Cases and Observations. By James Syme, Esq.

Case 1. Dislocation of the Shoulder-joint, of seven weeks standing; Reduction, - - - - - - - - - - - - - 309

Case 2. Dislocation of the Shoulder-joint, of seven weeks standing, reduced, - - - - - - - - - - - - - - 311
Case 3. Dislocation of the Shoulder-joint, of four weeks standing: Reduction, - - - - - - - - - - - 311
Case 4. Dislocation of the Shoulder-joint, of four weeks standing: Reduction, - - - - - - - - - - - 312
Case 5. Dislocation of the Hip-joint of five weeks standing:—Reduction, - - - - - - - - - - - 312
Case 6. Dislocation of the Thigh-bone, upwards and backwards, primarily, on the dorsum ilii, secondarily into the ischiatic notch:—Reduction, - - - - - - - - - - 312
Rumination in the Human Subject. By Robert Read, Veterinary Surgeon, Crediton, - - - - - - - - - 314
Malignant Tumor of the Inferior Maxilla, - - - - - - 315
Fibrous Tumor of the Inferior Maxilla, - - - - - - 316
Encysted Tumor in the Liver: Hydatids, - - - - - - 316
Process for the Detection of Arsenic, - - - - - - - 317
On the Administration of Intoxicating Doses of Alcohol in Traumatic Tetanus. By J. W. Stapleton, Trowbridge, - - - - 319
Autoplasty, - - - - - - - - - - - - 322

